# Suboptimal Activation of Antigen-Specific CD4^+^ Effector Cells Enables Persistence of *M. tuberculosis* In Vivo

**DOI:** 10.1371/journal.ppat.1002063

**Published:** 2011-05-26

**Authors:** Tyler D. Bold, Niaz Banaei, Andrea J. Wolf, Joel D. Ernst

**Affiliations:** 1 Department of Pathology, New York University School of Medicine, New York City, New York, United States of America; 2 Division of Infectious Diseases, Department of Medicine, New York University School of Medicine, New York City, New York, United States of America; 3 Department of Microbiology, New York University School of Medicine, New York City, New York, United States of America; University of Washington, United States of America

## Abstract

Adaptive immunity to *Mycobacterium tuberculosis* controls progressive bacterial growth and disease but does not eradicate infection. Among CD4^+^ T cells in the lungs of *M. tuberculosis*-infected mice, we observed that few produced IFN-γ without ex vivo restimulation. Therefore, we hypothesized that one mechanism whereby *M. tuberculosis* avoids elimination is by limiting activation of CD4^+^ effector T cells at the site of infection in the lungs. To test this hypothesis, we adoptively transferred Th1-polarized CD4^+^ effector T cells specific for *M. tuberculosis* Ag85B peptide 25 (P25TCRTh1 cells), which trafficked to the lungs of infected mice and exhibited antigen-dependent IFN-γ production. During the early phase of infection, ∼10% of P25TCRTh1 cells produced IFN-γ in vivo; this declined to <1% as infection progressed to chronic phase. Bacterial downregulation of *fbpB* (encoding Ag85B) contributed to the decrease in effector T cell activation in the lungs, as a strain of *M. tuberculosis* engineered to express *fbpB* in the chronic phase stimulated P25TCRTh1 effector cells at higher frequencies in vivo, and this resulted in CD4^+^ T cell-dependent reduction of lung bacterial burdens and prolonged survival of mice. Administration of synthetic peptide 25 alone also increased activation of endogenous antigen-specific effector cells and reduced the bacterial burden in the lungs without apparent host toxicity. These results indicate that CD4^+^ effector T cells are activated at suboptimal frequencies in tuberculosis, and that increasing effector T cell activation in the lungs by providing one or more epitope peptides may be a successful strategy for TB therapy.

## Author Summary

*Mycobacterium tuberculosis* causes persistent infection even in human or animal hosts that develop antigen-specific CD4^+^ and CD8^+^ T cell responses. To understand this phenomenon, we tested the hypothesis that the CD4^+^ effector T cells that are generated in response to *M. tuberculosis* infection fail to encounter their antigens at the site of infection in the lungs. Using mice infected with *M. tuberculosis*, and an assay of in vivo antigen-dependent activation of CD4^+^ T cells, we found that both polyclonal CD4^+^ and T cell receptor transgenic CD4^+^ T cells specific for antigen 85B peptide 25 are activated at low frequencies in the lungs. We found that this is due in part to downregulation of antigen gene expression by *M. tuberculosis*, as forced expression of the antigen gene resulted in higher frequency activation of CD4^+^ T cells, as well as CD4^+^ T cell-dependent reduction in bacterial burdens and prolonged survival of infected mice. We also found that administration of antigen 85B peptide 25, which is recognized by a high proportion of *M. tuberculosis*-specific CD4^+^ T cells, reduced the bacterial burden in the lungs, indicating that stimulation of existing antigen-specific CD4^+^ T cells may be a promising approach to therapy of TB.

## Introduction

Even though its etiologic agent was discovered over 125 years ago, tuberculosis remains a global scourge, killing 1.7 million people in 2009, at least ¾ of whom were immunocompetent [1]. Long-term persistence of *Mycobacterium tuberculosis*, which resides principally in phagocytic cells within the lungs, results in a chronic infection despite the presence of an apparently appropriate adaptive immune response. In mice infected with virulent *M. tuberculosis*, the early phase of infection proceeds with unchecked bacterial growth until day 17–21 post-infection, when adaptive immunity finally exerts control of bacterial growth in the lungs. Control of infection in both humans and mice critically depends on *M. tuberculosis*-specific CD4^+^ Th1 cell responses, which include production of IFN-γ [2], [3]; however adaptive immune responses do not eradicate the infection.

Several potential mechanisms may account for the failure of adaptive immune responses to eradicate the bacteria in tuberculosis. Generation of *M. tuberculosis*-specific CD4^+^ effector T cells is delayed compared with responses to other pathogens [2], [4]. In addition, certain individuals, or strains of mice, may develop inappropriate (*e.g.*, Th2) [5], [6] or imbalanced effector phenotypes such as Th1/Th17 [7] in response to infection. However, even in humans or mice that develop Th1 responses, a failure of CD4^+^ effector T cells to recognize infected cells may preclude their optimal activation and limit induction of effector functions in the lungs. Prevention of effector T cell activation could result from impaired antigen presentation by lung APCs containing *M. tuberculosis* [8], [9], [10] or because the antigens that effector T cells recognize are not expressed or otherwise available in the lungs. Furthermore, host regulatory mechanisms that limit immune pathology, such as T regulatory cells [11], production of inhibitory cytokines [12], and, possibly, onset of T cell exhaustion [13], [14] may inhibit the activity of effector T cells at the site of infection. Finally, even when CD4^+^ effector T cells are activated, the efficacy of these responses may be limited by the impaired ability of infected cells to respond to IFN-γ [15], [16], [17], induce phagosome maturation [18], [19], or undergo apoptosis [9], [20], [21], [22]. Understanding the contribution of each of these potential mechanisms limiting adaptive immunity to *M. tuberculosis* is an essential prerequisite for vaccine design and other immunologic approaches to tuberculosis prevention and therapy.

Here, we report that CD4^+^ effector T cells are activated at submaximal and suboptimal frequencies in the lungs during *M. tuberculosis* infection, that this is due in part to bacterial modulation of antigen expression, and that increasing the availability of a single antigen results in improved immune control of *M. tuberculosis*.

## Results

### Prevalence of CD4^+^ T cells activated to produce IFN-γ in the lungs of *M. tuberculosis*-infected mice

**Figure 1:**
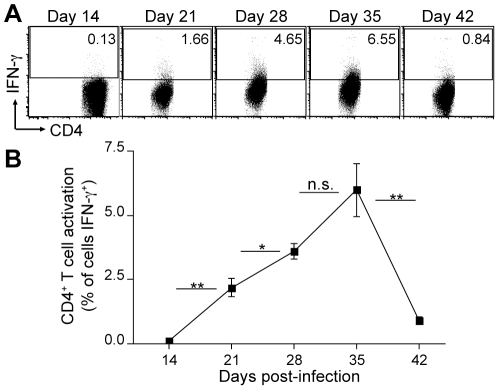
Low frequency of IFN-γ-producing endogenous CD4^+^ T cells in lungs of *M. tuberculosis*-infected mice. **A**. Frequency of IFN-γ expression by endogenous, polyclonal CD4^+^ T cells in the lungs of *M. tuberculosis*-infected mice throughout infection, assayed by intracellular cytokine staining without *ex vivo* restimulation. Flow cytometry dot plots show lung CD4^+^ cells from a representative mouse at the indicated time point post-infection. Values indicate the proportion of cells expressing IFN-γ in the CD4^+^ population for each mouse. **B**. Mean frequency of IFN-γ^+^ cells among lung CD4^+^ T cells for each group of 4 mice at each time point post-infection, assayed by intracellular cytokine staining without *ex vivo* restimulation. Asterisks indicate statistical significance of differences in frequency of T cell activation observed between adjacent time points * p<0.05; ** p<0.005.

We hypothesized that *M. tuberculosis* evades adaptive immunity by modulating the activation of CD4^+^ effector T cells at the site of infection in the lungs. Since in vitro studies have revealed evidence that *M. tuberculosis* modulates MHC class II antigen presentation [10], [23], [24], [25], [26], we focused on in vivo activation of CD4^+^ T cells in the lungs. We reasoned that, if *M. tuberculosis*-infected cells do not present antigens efficiently to effector T cells in the lungs, then the frequency of activation of effector functions of CD4^+^ cells would also be low at the site of infection. To test this, we used direct intracellular cytokine staining of lung cells from infected mice for IFN-γ, without ex vivo restimulation. We found that that the frequency of IFN-γ expression by CD4^+^ T cells in the lungs varied with the time of infection (Figure 1B). IFN-γ^+^ CD4^+^ cells were undetectable in the lungs at day 14, increased in frequency beginning by day 21 to a peak at day 35 post-infection, and then markedly decreased afterward; no more than 7% of the bulk population of CD4^+^ T cells expressed IFN-γ at any time point after infection, and fewer than 1% expressed IFN-γ during the chronic phase. Other studies investigating IFN-γ production by CD8^+^ T cells in vivo have used treatment of mice with brefeldin A or inclusion of brefeldin A during cell isolation and staining [27], [28]. However, we determined that these methods did not improve detection of intracellular IFN-γ by CD4^+^ T cells during *M. tuberculosis* infection (Figure S1). These data indicate that a small minority of polyclonal CD4^+^ T cells recruited to the lungs of *M. tuberculosis*-infected mice are activated to produce IFN-γ at a given time, and are consistent with defective antigen presentation, costimulation, and/or inhibition of effector T cell activation at the site of infection.

### Quantitating antigen-specific effector Th1 cell responses in the lungs of *M. tuberculosis*-infected mice

**Figure 2:**
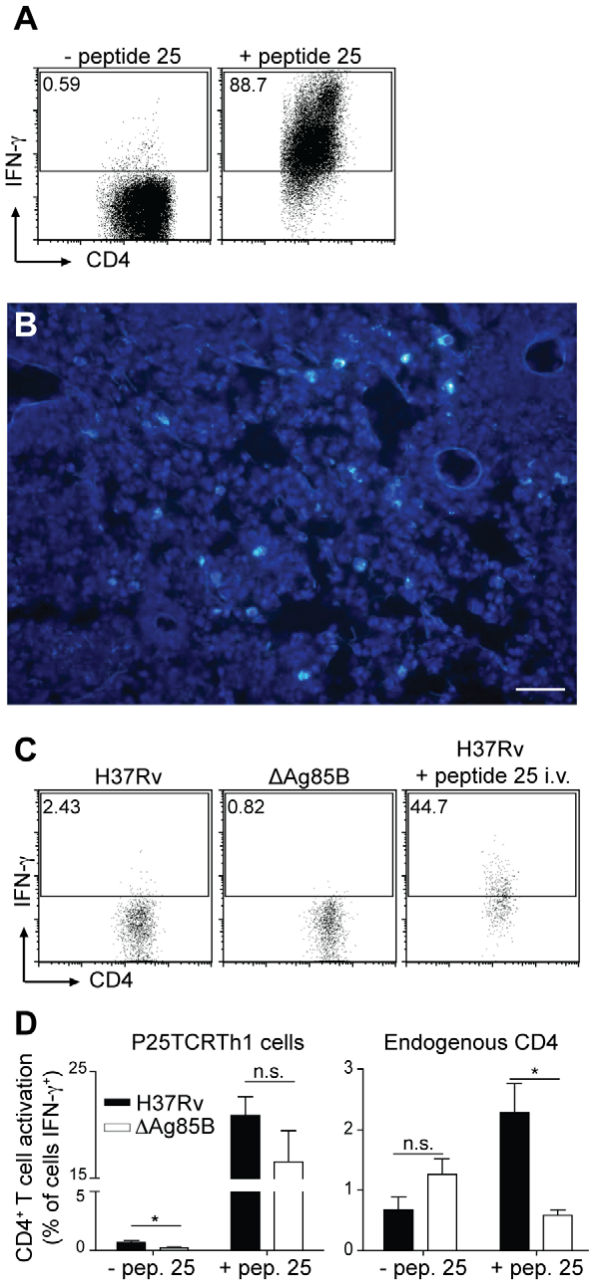
P25TCRTh1 cells produce IFN-γ in response to *M. tuberculosis* Ag85B peptide 25. **A**. P25TCRTh1 cells were restimulated in vitro with C57BL/6 splenocytes in the presence or absence of peptide 25 and analyzed by flow cytometry for intracellular IFN-γ. **B**. CFP-P25TCRTh1 cells traffic to the lung parenchyma of *M. tuberculosis*-infected mice. Th1 effector cells were transferred on day 25, and lungs were harvested on day 28 postinfection. CFP-P25TCRTh1 cells (light blue-green) are found in interstitial regions with a high density of DAPI-stained nuclei, typical of the aggregates of macrophages, dendritic cells, and lymphocytes observed at this stage of infection. Scale bar: 50 µm. **C**. On day 18 post-infection, mice infected with either H37Rv (w.t.) or ΔAg85B *M. tuberculosis* received P25TCRTh1 cells by adoptive transfer. Lung cells were harvested 72 hours later (day 21). Transferred P25TCRTh1 (CD45.2^+^) cells were analyzed by flow cytometry for intracellular IFN-γ. One group of mice received intravenous treatment with Ag85B peptide 25 6 hours prior to lung cell harvest. Flow cytometry dot plots from in vivo experiments show a representative of four mice per group. **D**. Day 21 post-infection with either H37Rv or ΔAg85B: mean percentage from four individual mice of P25TCRTh1 or endogenous CD4^+^ T cells expressing IFN-γ with or without in vivo administration of Ag85B peptide 25.

Since the low frequency of CD4^+^ T cell expression of IFN-γ in the lungs of *M. tuberculosis*-infected mice could be due to the presence of effector cells that traffic to the lungs but are not specific for *M. tuberculosis* antigens, we performed the remainder of our studies using CD4^+^ TCR transgenic T cells that specifically recognize a well-characterized immunodominant *M. tuberculosis* antigen. To quantitate the frequency of activation of *M. tuberculosis* antigen-specific effector cells in the lungs, we prepared CD4^+^ Th1 effector cells (P25TCRTh1 cells) from transgenic mice with a TCR specific for peptide 25 (amino acids 240–254) of Ag85B. When P25TCRTh1 cells were incubated with irradiated splenocytes in the absence of peptide 25, <1.0% of the cells expressed IFN-γ as detected by intracellular staining and flow cytometry, whereas addition of peptide 25 in vitro induced IFN-γ expression in ∼90% of cells (Figure 2A). This result demonstrated that the frequency of IFN-γ staining in P25TCRTh1 cells can specifically assay antigen dependent stimulation of P25TCRTh1 cells.

### P25TCRTh1 cells recognize antigen at low frequency in vivo

Since Day 21 post-infection corresponds to an acute stage of infection when adaptive immune effector mechanisms have been initiated and reduce the rate of bacterial population growth in the lungs, and since it resembles the stage of LCMV infection in which a high frequency of antigen-specific CD8^+^ T cell responses are observed [28], we chose this time point for initial characterization of P25TCRTh1 cell responses in vivo. We verified that adoptively transferred P25TCRTh1 cells traffic to the site of infection by examining sections of lungs from infected mice that had received CFP^+^ P25TCRTh1 cells. CFP^+^ cells were abundant in the lung parenchyma, and were concentrated in granulomas (Figure 2B). Furthermore, we determined that >85% of the transferred cells were protected from labelling by an i.v. injection of PerCP-labeled anti-CD4 antibody, indicating that adoptively transferred P25TCRTh1 cells efficiently migrate out of the lung vasculature into the parenchyma of infected lungs (Figure S2A).

To determine the frequency of activation of antigen-specific CD4^+^ effector T cells in the lungs early in infection, we adoptively transferred P25TCRTh1 cells on day 18 and harvested them on day 21 after infection of wild-type mice with wild-type *M. tuberculosis* H37Rv. The frequency of IFN-γ^+^ P25TCRTh1 cells isolated from the lungs was unexpectedly low at Day 21 post-infection (Figure 2C and 2D). Approximately 1–2% of the transferred P25TCRTh1 cells were stimulated to produce IFN-γ in vivo at that time point (Figure 2C), and this percentage was similar to the frequency of total endogenous lung CD4^+^ T cells expressing IFN-γ on day 21 post-infection (Figure 1B, 2D). Moreover, after intravenous injection of PerCP-labeled anti-CD4 antibody, the only IFN-γ^+^ P25TCRTh1 cells identified were PerCP negative (Figure S2B), indicating that the responding cells were those that had migrated out of the vasculature into the lung parenchyma and were protected from staining by the in vivo injection of antibody.

We verified that stimulation of P25TCRTh1 cells to express intracellular IFN-γ is due to recognition of Ag85B peptide 25 by transferring P25TCRTh1 cells into mice infected with an Ag85B-null strain of *M. tuberculosis* (ΔAg85B), which is equivalent to wild-type H37Rv in virulence [2]. A lower mean percentage (0.74%) of P25TCRTh1 cells isolated from ΔAg85B-infected mice expressed IFN-γ than those from H37Rv-infected mice (Figure 2C and 2D). This indicates that in vivo IFN-γ production by P25TCRTh1 cells is antigen-dependent and not the consequence of inflammatory cytokines present at the site of infection. We also evaluated several alternative approaches to detecting effector T cell activation in the lungs. P25TCRTh1 cells expressed both CD25 and CD44 prior to adoptive transfer, which excluded their use in evaluating effector cell activation in vivo. Surface expression of CD69 was induced after adoptive transfer of P25TCRTh1 effector cells into H37Rv-infected mice; however, we found similar induction of CD69 in mice infected with ΔAg85B, indicating that it did not specifically reflect antigen-dependent effector cell activation. This result, together with evidence that CD69 can be induced by costimulation and by certain cytokines present at the site of *M. tuberculosis* infection [29], [30], [31], indicates that expression of intracellular IFN-γ is the most accurate reporter of antigen specific Th1 effector cell activation in the lungs. Together, these results indicate that even though they traffic efficiently to the site of infection, Ag85B peptide 25-specific CD4^+^ effector cells are activated to execute their Th1 effector function at low frequency in the lungs of *M. tuberculosis*-infected mice.

### P25TCRTh1 cells are capable of responding to antigen at the site of infection

Although IFN-γ production by P25TCRTh1 cells at day 21 was dependent on Ag85B, the frequency of IFN-γ^+^ cells was surprisingly low in H37Rv infected mice. One possible explanation for the low frequency of activation of effector cells is that their cognate antigen is not available for recognition at the site of infection. To test this hypothesis, we provided antigen in vivo by injecting peptide 25 intravenously into mice that had been infected 21 days earlier. When P25TCRTh1 recipient, H37Rv-infected mice received peptide 25 six hours prior to lung cell harvest, the frequency of IFN-γ^+^ P25TCRTh1 cells increased to 20–50% (Figure 2C and 2D). Similarly, peptide 25 injection stimulated a higher frequency of IFN-γ expression by endogenous CD4^+^ T cells from mice infected with H37Rv (Figure 2C and 2D), consistent with prior evidence that peptide 25 of Ag85B is a dominant antigen in C57BL/6 mice infected with *M. tuberculosis* [32], [33]. P25TCRTh1 cells transferred into ΔAg85B-infected recipients were also stimulated at a higher frequency after intravenous peptide 25 treatment, while endogenous CD4^+^ T cells from ΔAg85B-infected mice did not respond to peptide 25 with increased IFN-γ expression (Figure 2D). The failure of endogenous CD4^+^ T cells from ΔAg85B-infected mice to respond to peptide 25 injection reflects the absence of Ag85B peptide 25-specific effector T cells generated in response to this infection. These results indicate that the frequency of IFN-γ^+^ P25TCRTh1 cells is an accurate and specific measure of CD4^+^ effector T cell stimulation in response to presentation of Ag85B peptide 25 in vivo. The observation that in vivo IFN-γ responses to peptide 25 injection depend on the presence of previously-generated (endogenous or transferred) peptide 25-specific effector T cells indicates that the responses are not due to a nonspecific effect of the epitope peptide on costimulation or responses of CD4^+^ T cells with specificity for other antigens. In addition, they demonstrate that if antigen is made available to them, adoptively transferred P25TCRTh1 cells can respond to antigen in the infected lungs, and they provide evidence against an exclusive role for T regulatory cells and/or suppressive cytokines in limiting the activation of CD4^+^ effector cells at the site of *M. tuberculosis* infection in the lungs.

To further characterize the in vivo assay system, and to evaluate the possibility that low frequencies of P25TCRTh1 responses are attributable to either competition for antigen by endogenous CD4^+^ T cells and/or a dominant effect of T regulatory cells, we specifically ablated endogenous T cells from *M. tuberculosis*-infected CD4-DTR mice [34] prior to assaying P25TCRTh1 responses in vivo. Compared to untreated mice, DT treatment reduced the fraction of endogenous CD4^+^ T cells in the lung by an average of 48.9%, p = 0.0053 (Figure S3A). However, this had no effect on the percentage of P25TCRTh1 cells activated to produce IFN-γ (Figure S3B). These results strongly suggest that the low frequency of activation of P25TCRTh1 cells is caused neither by competition for peptide 25:MHC II complexes by endogenous CD4^+^ T cells, nor by the influence of T regulatory cells in the lungs. We therefore conclude that the response of adoptively transferred P25TCRTh1 cells is an accurate reflection of MHC II presentation of Ag85B peptide 25 by lung APCs during infection.

### Dynamics of *M. tuberculosis*-specific CD4^+^ effector T cell responses during the course of infection

**Figure 3:**
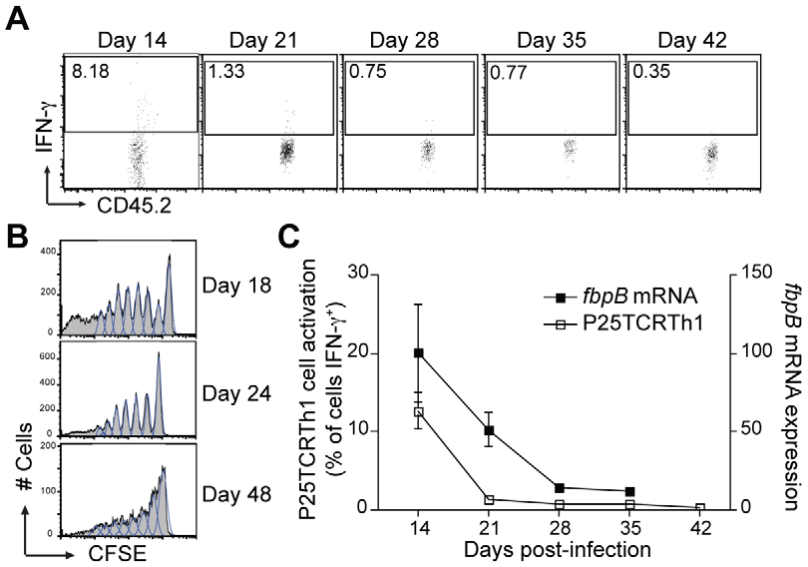
Peptide 25-specific T cell activation and *fbpB* expression decrease during chronic infection. **A**. Frequency of IFN-γ production by adoptively transferred P25TCRTh1 (CD45.2^+^) CD4^+^ cells in the lungs of *M. tuberculosis*-infected mice throughout infection, assayed by intracellular cytokine staining without *ex vivo* restimulation. Flow cytometry dot plots show lung P25TCRTh1 cells which were adoptively transferred 3 days prior to the indicated time point post-infection. Values indicate the proportion of cells expressing IFN-γ among the CD45.2^+^, CD4^+^ population for each mouse. **B**. CFSE proliferation profile of naïve P25TCR-tg CD4^+^ T cells transferred into *M. tuberculosis*-infected wild type recipients on days 11, 17 or 35 post-infection. Mediastinal lymph node cells were isolated 7 days after adoptive transfer (days 18, 24, or 48 post-infection) and analyzed by flow cytometry for CFSE dilution to measure proliferation. Histograms are representative of four individual mice per time point. **C**. The mean percentage of P25TCRTh1 cells from four individual mice expressing IFN-γ at each time point post-infection is compared with the expression of *M. tuberculosis fbpB* mRNA as infection progresses to chronic phase. Copy number of *fbpB* mRNA for four individual mice at each time point was determined by RT-qPCR and is normalized to constitutively expressed 16S rRNA.

Adaptive immunity restricts progressive growth of *M. tuberculosis*, but it does not eliminate the bacteria from the lungs, which results in chronic infection in mice and latent infection in humans. To determine whether suboptimal activation of *M. tuberculosis*-specific T cells contributes to the ability of the bacteria to persist, we first asked whether activation of P25TCRTh1 cells in the lungs changes as infection progresses to a chronic phase. To compare the frequency of effector T cell stimulation at various stages of infection, we transferred P25TCRTh1 cells into H37Rv-infected mice on day 11, 18, 25, 32, or 39 post-infection. Lung cells were harvested 72 hours after transfer (day 14, 21, 28, 35, or 42 post-infection) and analyzed by flow cytometry for intracellular IFN-γ without ex vivo restimulation. The proportion of P25TCRTh1 cells producing IFN-γ was highest (∼10%) on day 14 (Figure 3A and 3C). These results indicate that during the acute stage of infection, adoptively transferred P25TCRTh1 cells are stimulated in the lungs at a frequency comparable to that of TCR transgenic CD4^+^ effector cells at the site of injection of a protein antigen and adjuvant [35]. In contrast, expression of IFN-γ by endogenous (CD45.2^−^) CD4^+^ cells was rare (<0.1%) at that time point (Figure 1B and 1C). The difference between transferred and endogenous cell responses on day 14 is consistent with our previous observation that initiation of adaptive immunity to *M. tuberculosis* is delayed until day 11–14 post-infection, and consequently, endogenous CD4^+^ effector T cells specific for *M. tuberculosis* antigens are first detected in the lungs on day 17 post-infection. [2].

The frequency of IFN-γ production by P25TCRTh1 cells progressively decreased from day 14 to day 42 post-infection, indicating a decrease in the efficiency of peptide 25-specific T cell stimulation as infection enters its chronic phase (Figure 3A and 3C). These results with TCR transgenic CD4^+^ effector cells closely mimic the results observed with endogenous polyclonal CD4^+^ T cells after day 14 post-infection (Figure 1B and 1C). Although Ag85B peptide 25-specific responses reached an earlier peak and decreased earlier than did those of endogenous polyclonal CD4^+^ T cell responses, the results with the two cell populations were similar, with endogenous CD4^+^ effector T cell responses also diminishing by day 42 post-infection.

To determine whether activation of naïve Ag85B peptide 25-specific CD4^+^ T cells is also diminished in the later stages of *M. tuberculosis* infection, we assayed the response of adoptively transferred naïve P25 TCR-Tg T cells in the lung-draining mediastinal lymph nodes of H37Rv-infected mice at various time points post-infection. 7 days after transfer, we harvested lymph node cells and measured in vivo T cell proliferation by flow cytometry using a CFSE dilution assay. The rate of naïve P25TCR-tg T cells was highest upon transfer into mice on day 18 post-infection, while fewer cells exhibited CFSE dilution at days 24 and 48 post-infection (Figure 3B). These results indicate that decreased stimulation of P25TCRTh1 effector cells is also accompanied by decreased generation of peptide 25 specific effector T cells from naive cells at later stages of infection.

### Progressive decreases of P25TCRTh1 cell activation accompany decreased *fbpB* expression

Since treatment of infected mice with exogenous peptide 25 enhanced T cell responses, indicating that adoptively-transferred P25TCRTh1 cells are capable of responding to antigen stimulation in the lungs, we hypothesized that availability and/or presentation of antigen is a limiting factor in the activation of CD4^+^ effector T cells at the site of *M. tuberculosis* infection. To test this hypothesis, we first investigated whether changes in the expression of the *M. tuberculosis* gene that encodes Ag85B influence the frequency of activation of P25TCRTh1 effector cells. We found that the frequency of in vivo activation of P25TCRTh1 cells mimicked the temporal pattern of expression of *fbpB* (which encodes Ag85B) by *M. tuberculosis* in vivo (Figure 3C). This suggests that reduced expression of Ag85B contributes to the low frequency of activation of Ag85B-specific CD4^+^ effector cells in the lungs, thus resembling previously-reported observations with Salmonella FliC expression and FliC-specific CD4^+^ T cell responses [36].

### Forced expression of *fbpB* induces greater P25TCRTh1 cell activation

**Figure 4:**
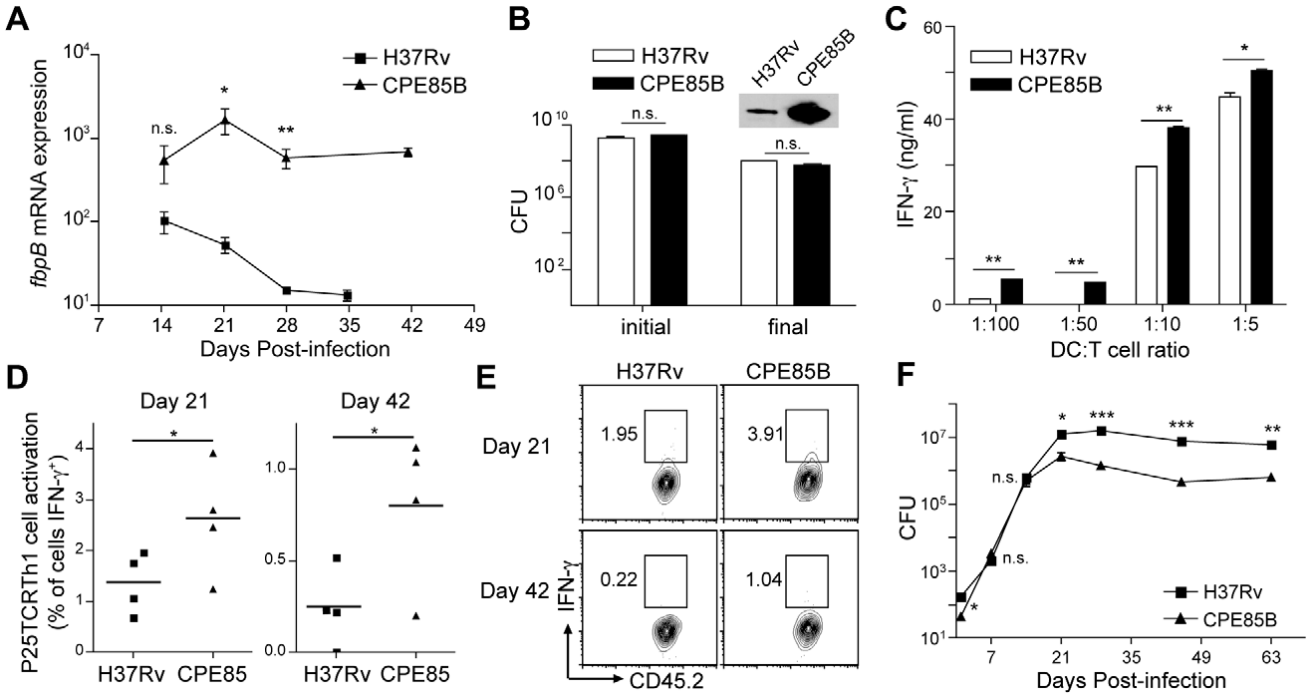
Forced expression of *fbpB* enhances T cell activation and impairs bacterial persistence during chronic infection. **A**. Expression of *fbpB* mRNA, normalized to 16S rRNA by H37Rv and CPE85B throughout in vivo infection, determined by RT-qPCR of bacteria in lungs. Data points indicate the mean (±SEM) of 4 mice per time point. **B**. Bacterial population size of H37Rv or CPE85B *in vitro* culture before and after stationary liquid incubation to induce expression of *hspXp:fbpB*. Columns represent the mean (±SEM) population size of three cultures for each strain. Western blot shows Ag85B protein secreted into culture supernatants during stationary culture. **C**. Activation of P25TCRTh1 cells *in vitro* by bone marrow derived DCs infected with H37Rv or CPE85B, measured by IFN-γ ELISA. Columns represent the mean (±SEM) of 3 wells at the indicated infected DC∶T cell ratio. **D**, **E**. Activation of P25TCRTh1 cells during mouse infection with H37Rv or CPE85B. P25TCRTh1 cells were transferred into infected mice on either day 18 or 39 post-infection. 3 days after adoptive transfer (day 21 or 42), lung cells were analyzed by flow cytometry for intracellular IFN-γ without *ex vivo* restimulation. (**D**) Data points indicate the frequency of cells expressing IFN-γ among CD45.2^+^, CD4^+^ lung cells at each time point. (**E**) Flow cytometry plots show lung P25TCRTh1 cells from a representative mouse at the indicated time point post-infection. Values indicate the proportion of IFN-γ^+^ cells among CD45.2^+^, CD4^+^ population. **F**. Bacterial population size throughout mouse infection with H37Rv or CPE85B. Data points indicate the mean (±SEM) of 4 mice per time point.

To test the hypothesis that *fbpB* down-regulation contributes to the submaximal frequency of CD4^+^ effector cell activation and the limited efficacy of the Th1 response in vivo, we constructed a recombinant strain of *M. tuberculosis* to express *fbpB* at high levels during chronic infection. Using the ΔAg85B strain as a background, we introduced a wild-type *fbpB* allele under control of the *hspX/acr/Rv2031c* promoter to the *M. tuberculosis* chromosome via the pMV306 integrating vector. *hspX* is expressed at high levels during chronic phase infection in an expression pattern inverse to *fbpB* [37], [38]. This strain (*hspXp:fbpB*, termed “CPE85B” for chronic phase expressed Ag85B) exhibited higher *fbpB* expression compared to H37Rv in the lungs of mice after aerosol infection (Figure 4A). The expression of *fbpB* measured by RT-qPCR was approximately 10-fold higher (normalized for the abundance of 16S rRNA) at day 21 post-infection for CPE85B than for H37Rv. As the infection progressed to chronic phase (day 28–42 post-infection), *fbpB* expression from the native promoter declined by approximately 100-fold while *fbpB* expression driven by the *hpsX* promoter remained at nearly constant, higher levels (Figure 4A). Increased *fbpB* gene expression in the CPE85B strain was accompanied by markedly enhanced expression and secretion of Ag85B protein when the *hspX* promoter was induced in stationary liquid culture (Figure 4B).

To determine whether forced expression of *fbpB* in *M. tuberculosis* results in increased presentation of Ag85B peptide 25 to CD4^+^ T cells, we infected bone marrow-derived dendritic cells (BMDC) with either H37Rv or CPE85B and compared their ability to activate P25TCRTh1 cells in culture. At all APC∶T cell ratios examined, DCs infected with CPE85B induced significantly greater amounts of IFN-γ secretion from P25TCRTh1 cells than did DCs infected with H37Rv (Figure 4C). To determine whether forced expression of *fbpB* can increase the frequency of P25TCRTh1 stimulation during H37Rv infection in vivo, we compared the frequency of P25TCRTh1 cell activation in the lungs of mice infected with either H37Rv or CPE85B. Compared to cells from H37Rv-infected recipients, P25TCRTh1 cells from CPE85B-infected mice produced IFN-γ with a 2-fold (day 21) to 5-fold (day 42) higher frequency (Figure 4D and E). These findings indicate that forced expression of *fbpB* by *M. tuberculosis* increases the proportion of P25TCRTh1 cells that are activated to produce IFN-γ in the lungs. By suppressing *fbpB* expression after the initial stages of infection, wild-type *M. tuberculosis* can reduce the frequency of activation of Ag85B-specific effector T cells. Although expression of *fbpB* was maintained at high levels from day 14 to day 42 post-infection, P25TCRTh1 cell stimulation in CPE85B-infected mice was only two- to five-fold higher than in mice infected with H37Rv, and decreased as infection progressed to chronic stage, indicating that other mechanisms, such as inhibition of antigen presentation and/or induction of regulatory T cells, exist to limit the activation of CD4^+^ effector T cells in the lung.

### Forced expression of *fbpB* impairs bacterial persistence during chronic infection

We reasoned that, if diminishing *fbpB* expression during chronic infection limits effector T cell activation and thereby enables *M. tuberculosis* to evade adaptive immunity, then constitutive expression of *fbpB* throughout infection should improve immune control of infection. To test this hypothesis, we infected mice with either H37Rv or CPE85B and quantitated *M. tuberculosis* CFUs in the lungs throughout the course of infection. The rates of bacterial growth for the two strains were indistinguishable prior to day 14 post infection (Figure 4F), indicating that expression of *fbpB* by the *hspX* promoter does not attenuate *M. tuberculosis* in vivo during the innate immune stage of infection, prior to recruitment of CD4^+^ effector T cells to the lungs. Indeed, the in vivo generation time of the CPE85B strain (23.0 h) was slightly shorter than that of H37Rv (26.4 h) during days 1–14 of infection (these are not significantly different by nonlinear curve fit and *F* test). However, at times corresponding to the adaptive immune phase of infection, the bacterial burden of the CPE85B strain in the lungs was approximately 10-fold lower than that of H37Rv (Figure 4F). These results suggest that forced expression of *fbpB* partially overcomes the antigen deficit that limits the activation of CD4^+^ T cells in the lung during chronic infection and allows greater antimycobacterial efficacy of the adaptive immune response.

### Chronic phase attenuation of CPE85B is dependent on CD4^+^ T cells

**Figure 5:**
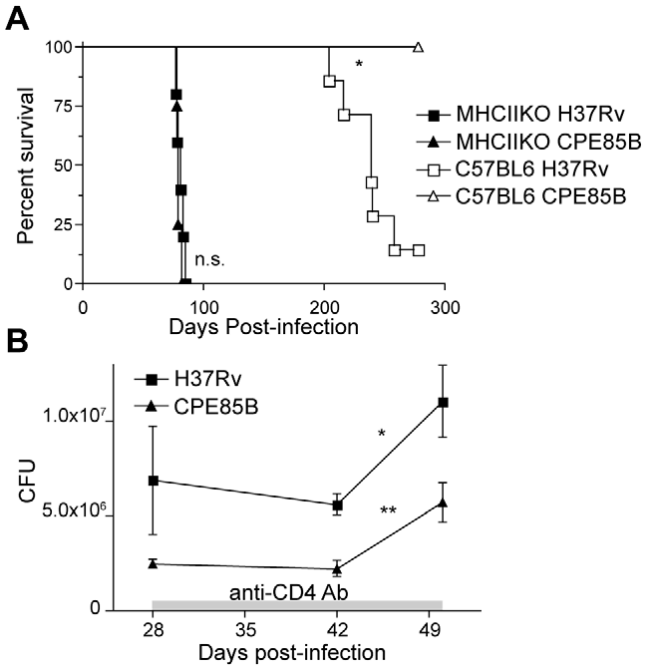
Forced expression of *fbpB* impairs *M. tuberculosis* in a CD4^+^ T cell dependent manner. **A**. Survival of C57BL/6 and CD4^+^ T cell-deficient MHCII KO mice after aerosol infection with H37Rv or CPE85B. N≥5 mice for each group. **B**. Bacterial population size in lungs of mice infected with H37Rv or CPE85B after CD4^+^ T cell depletion with monoclonal anti-CD4 antibody GK1.5. Antibody treatment was started on day 28 post-infection and continued every 6 days until day 50. Data points indicate the mean (±SEM) bacterial burden in 4 mice in each infection group at each time point. Asterisks indicate statistical significance between groups of mice at neighboring time points within one infection group; * p<0.05; ** p<0.005.

The observation that CPE85B demonstrates a growth pattern indistinguishable from H37Rv during the first two to three weeks of infection, prior to onset of adaptive immunity, suggested that CPE85B was not inherently attenuated for growth in vivo. However, we considered the possibility that over-expression of *fbpB* could cause attenuation of *M. tuberculosis* as a result of gene dysregulation or toxicity of an overabundant Ag85B protein. Notably CPE85B demonstrated a similar growth pattern to H37Rv during in vitro shaking culture. Furthermore, under conditions of hypoxic stationary culture, when Ag85B protein is strongly expressed by CPE85B compared to H37Rv, the survival of the CPE85B strain is not impaired compared with that of wild-type bacteria (Figure 4B). Taken together, these findings imply that impaired persistence of *M. tuberculosis* CPE85B in vivo is the consequence of increased antigen presentation and activation of CD4^+^ T cells, and not due to intrinsic attenuation of the CPE85B strain in vitro or in vivo. We reasoned that if the decreased lung bacterial burden of CPE85B compared with that of H37Rv is attributable to increased antigen presentation and recognition by CD4^+^ T cells, then the attenuated phenotype of CPE85B should be abrogated in mice lacking CD4^+^ T cells. Indeed, whereas wild type C57BL/6 mice infected with CPE85B survived significantly longer than those infected with H37Rv (median survival >300 and 239 days, respectively; p = 0.0062), MHCIIKO mice, which lack CD4^+^ T cells, exhibited indistinguishable susceptibility to infection with the CPE85B and H37Rv strains (median survival 79 and 81 days, respectively; p = 0.425), (Figure 5A), clearly establishing that in vivo attenuation of the CPE85B strain depends on MHC II antigen presentation and CD4^+^ T cell responses. These results also indicate that increased antigen expression, accompanied by increased antigen-specific T cell activation, can enhance control of *M. tuberculosis* without detectable detrimental effects, since wild-type mice infected with the CPE85B strain survived longer than mice infected with H37Rv.

Since MHC II-deficient mice are highly susceptible to *M. tuberculosis* infection, this could potentially mask any hypothetical CD4^+^ T cell-independent mechanisms of attenuation of the CPE85B strain. We reasoned that, if mechanisms other than increased CD4^+^ T cell recognition contribute to the lower burdens of CPE85B, then this strain would not recover and grow normally in the lungs when CD4^+^ T cells are depleted during the chronic phase of infection. We infected mice with H37Rv or CPE85B and allowed the infection to proceed for 28 days, when initial lung CFUs were measured for each group. As expected, bacterial CFUs for CPE85B were ∼3 fold lower than H37Rv at this time point (Figure 5B). The remaining mice in each infection group were then treated with monoclonal antibody GK1.5 every 6 days until day 50 post-infection to deplete CD4^+^ T cells. After an initial lag, in which neither bacterial strain expanded, both CPE85B and H37Rv resumed growth in the lungs at indistinguishable rates (Figure 5B). Taken together, these data provide strong evidence that improved control of the CPE85B strain is attributable to increased activation of Ag85B-specific CD4^+^ T cells, although we cannot exclude the possibility that other factors contribute to the lower lung burdens of CPE85B that appear after the development of adaptive immunity.

### Treatment with intravenous peptide 25 during chronic infection reduces lung bacterial burden

**Figure 6:**
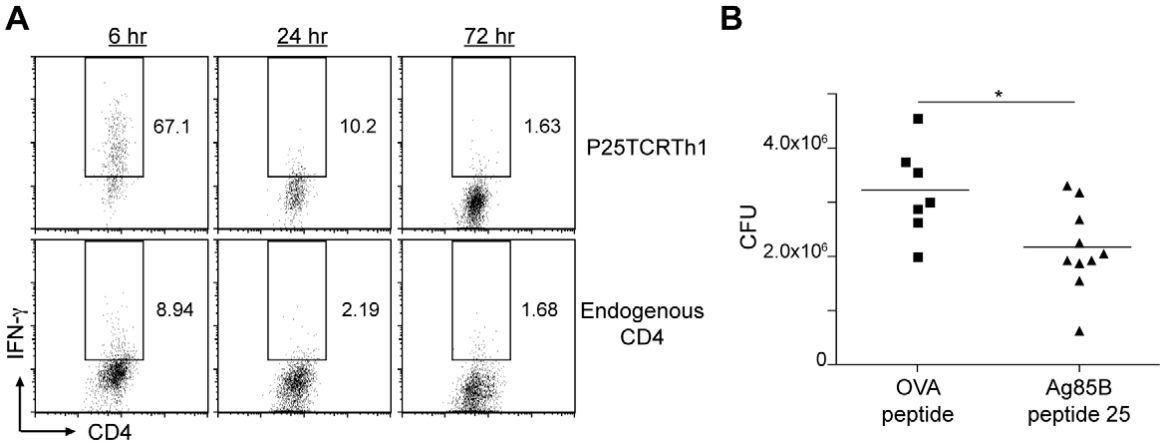
Treatment with peptide 25 transiently enhances CD4^+^ T cell responses and reduces bacterial burden. (**A**) Frequency of adoptively transferred P25TCRTh1 (top row) or endogenous (bottom row) CD4^+^ T cells producing IFN-γ at various time points after intravenous treatment with synthetic Ag85B peptide 25. Flow cytometry dot plots show lung CD4^+^ cells from a representative mouse at the indicated time point after treatment with peptide 25. Values indicate the proportion of IFN-γ^+^ cells among the CD45.2^+^ or CD45.2^−^, CD4^+^ population for each mouse. Data shown are representative of n≥4 mice per group. (**B**) Bacterial burden in the lungs of wild type mice treated from day 28 to day 45 post-infection with intravenous Ag85B peptide 25 or OVA peptide control. Data points indicate the final bacterial population size for individual mice in each group after treatment with either peptide. Data shown are representative of n≥4 mice per group.

Our observation that forced expression of *fbpB* increased the frequency of Ag85B peptide 25-specific CD4^+^ T cells and reduced the bacterial burden in the lungs (Figure 4D, 4E, and 4F), together with our observation that injection of peptide 25 also increased activation of CD4^+^ effector T cells at the site of infection (Figure 2B and 2C) suggested that providing antigen by injection of peptide 25 might also result in improved immune control of infection. We first determined the duration of increased IFN-γ production by adoptively transferred P25TCRTh1 cells or endogenous CD4^+^ T cells after peptide 25 injection. The frequency of IFN-γ cells was highest in both 6 hours after treatment, and decreased to approximately 20% of maximal levels by 24 hours after peptide injection for both endogenous CD4^+^ and P25TCRTh1 cells (Figure 6A). By 72 hours post-treatment, the frequency of IFN-γ^+^ cells returned to levels observed in the absence of peptide 25 injection, indicating that the activating effect of peptide 25 treatment is remarkably transient, entirely dissipating within 3 days of the treatment. Despite the transient nature of this effect, we found that treatment of *M. tuberculosis* H37Rv-infected mice with peptide 25 (in the absence of adoptively transferred P25TCRTh1 cells) every 2–3 days from day 28 to day 45 post-infection reduced lung bacterial burdens by 1.05±0.40×10^6^ bacteria (p = 0.018) compared with that in mice treated with OVA peptide, an unrelated MHC II epitope (Figure 6B). Neither group of mice displayed any signs of toxicity, even after repeated peptide injections. These results indicate that during *M. tuberculosis* infection, CD4^+^ effector T cells are not stimulated at their maximum potential frequency at the site of infection in the lungs. Because effector T cell responses progressively decrease during chronic infection, and enhancing T cell responses with exogenous peptide antigen improves immune clearance of *M. tuberculosis*, we conclude that failure to optimally activate effector T cells at the site of infection is an important determinant of the limited efficacy of adaptive immunity in tuberculosis.

## Discussion

**Figure 7:**
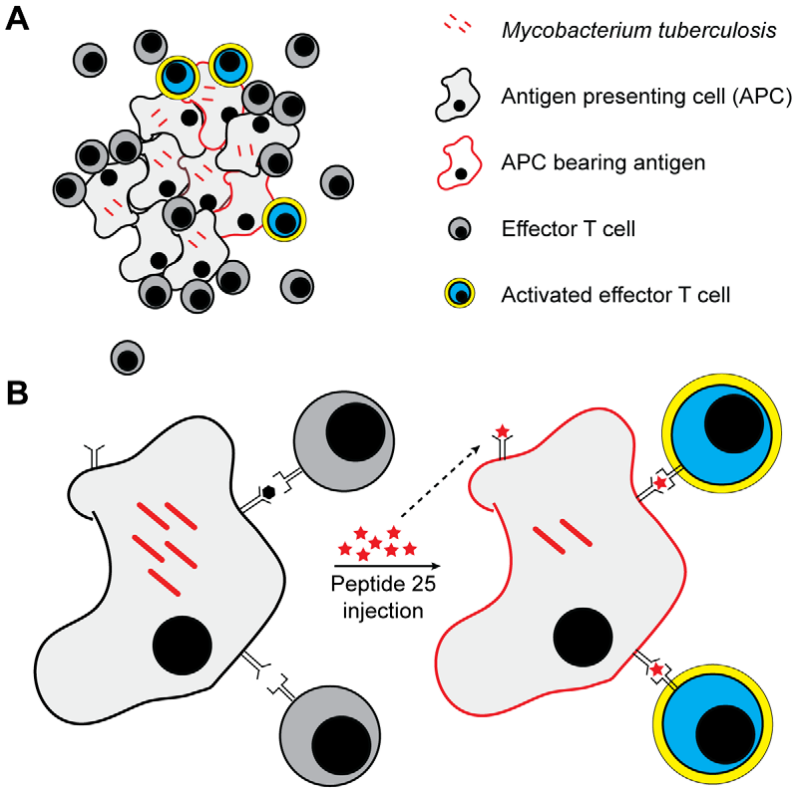
Schematic diagram of CD4^+^ effector T cell activation at the site of *M. tuberculosis* infection. **A**. During the chronic stage of infection, Ag85B-specific CD4^+^ effector cells are activated at low frequencies, at least part due to low bacterial expression of the antigen gene; bacteria are able to persist due to the low frequency of effector cell activation. **B**. Administration of epitope peptide occupies previously-empty MHC class II and/or displaces previously-bound peptides and provides antigen for recognition by pre-existing epitope-specific CD4^+^ effector cells, resulting in their activation and consequent reduction of the lung bacterial burden.

*M. tuberculosis* evades adaptive immunity to persist in the lungs, often for the lifetime of the host. Here, we have characterized one mechanism by which this impressive feat of immune evasion is accomplished in vivo. We found that, of the large number of CD4^+^ effector T cells recruited to the lungs of infected mice, few are stimulated to produce IFN-γ (Figure 7A). While there are few precedents available for comparison, our findings are in stark contrast to those found in C57BL/6 mice infected with the Armstrong strain of LCMV [28]. In that context, which results in CD8^+^ T cell-dependent resolution of infection, >20% of virus-specific CD8^+^ T cells are activated to produce IFN-γ during the acute stage of infection when viral burdens and antigen availability are highest, and the frequency of in vivo-activated virus-specific CD8^+^ T cells does not decrease until the viral burden is reduced. We found that the initially low proportion of CD4^+^ T cells producing IFN-γ in the lungs of *M. tuberculosis*-infected mice diminishes further as infection progresses to chronic phase, even though the bacterial burden in the lungs remains high. Our studies using adoptively transferred Ag85B-specific P25TCRTh1 cells revealed that the decreasing responses of CD4^+^ effector cells are caused in part by decreasing expression of *fbpB* by *M. tuberculosis*. By reducing *fbpB* expression during chronic infection, *M. tuberculosis* restricts the availability of Ag85B, an immunodominant antigen, and thereby prevents infected APCs from optimally activating CD4^+^ effector T cells. Consistent with this model, we found that a recombinant strain of *M. tuberculosis* engineered to maintain the expression of *fbpB* at high levels during chronic infection (CPE85B) was attenuated during the chronic phase of infection in a strictly CD4^+^ T cell dependent manner, indicating that down-regulation of *fbpB* and limitation of antigen availability is important for evasion of adaptive immunity by *M. tuberculosis*. Treatment of infected mice with synthetic Ag85B peptide 25 also increased CD4^+^ effector T cell IFN-γ responses and significantly reduced the bacterial burden in the lungs. We conclude that suboptimal effector T cell activation enables *M. tuberculosis* to evade elimination by adaptive immunity during the chronic stage of infection, and that some of this suboptimal effector T cell activation is attributable to restricted antigen expression by the bacteria. In addition, other mechanisms that limit effector T cell activation, such as interference with the MHC class II antigen processing and presentation pathway and/or the action of regulatory T cells, likely contribute to the remarkable survival of *M. tuberculosis* in vivo.

Infection with *M. tuberculosis* induces a robust T cell response involving CD4^+^ and CD8^+^ T cells and the effector cytokines IFN-γ and TNF [3], which are all essential for control of infection [5], [39], yet adaptive immunity fails to eradicate *M. tuberculosis*. Mechanisms for the limited efficacy of the adaptive immune response in tuberculosis fall into two general (not mutually exclusive) categories: either the effector functions that T cells perform (*e.g.* IFN-γ production) are not effective because of failed responses by the infected cells targeted by effector T cells; or the T cells recruited to the site of infection do not optimally perform the effector functions required for immune clearance. Regarding the former, the ability of *M. tuberculosis* to resist and inhibit the TNF- and IFN-γ-induced microbicidal responses of the phagocytic cells it infects is one documented component of its immune evasion strategy in vivo [40]. However, our observation that only a small fraction of the CD4^+^ effector T cells in the lungs is activated to synthesize IFN-γ provides new support for the latter explanation. The potential causes of this mechanism include bacterial factors and host regulatory mechanisms that directly impair effector T cell function. As an example of a direct bacterial effect, mycobacterial cell wall glycolipids have been found to impair CD4^+^ T cell responses in vitro [41]. With regard to host regulatory mechanisms, during mouse infection, T regulatory cells limit the ability of adaptive immunity to restrict the bacterial population size in the lungs [11], [42]. Interleukin-10 (IL-10), whether expressed by myeloid cells or T cells, provides an additional host regulatory mechanism that inhibits T cell effector functions in tuberculosis, as transgenic over-expression of IL-10 in infected mice impaired T cell responses and caused an increase in bacterial CFUs [12], while deletion of IL-10 causes enhanced control of infection [43], indicating that T cell-directed suppressive factors can limit the success of the adaptive immune response to *M. tuberculosis*. On the other hand, CD4^+^ effector T cells at the site of infection may not recognize or become activated optimally by APCs bearing *M. tuberculosis*-derived peptide:MHC II complexes, a process that is required for IFN-γ production in peripheral tissues [35]. Recent observations using live imaging revealed that a small fraction of *Leishmania major*-infected macrophages interact with *Leishmania*-specific CD4^+^ T cells in vivo [44] indicating that in certain infections, effector T cells may not recognize infected cells efficiently, and this may contribute to slow clearance or persistence of infection. Suboptimal stimulation of CD4^+^ T cells could occur via direct targeting and inhibition of MHC II antigen presentation pathways in infected APCs, or as a result of the limited availability of peptide T cell epitopes, a consequence of bacterial suppression of antigen encoding genes, or a combination of these mechanisms.

In this study, we first determined that the frequency of endogenous polyclonal CD4^+^ T cells producing IFN-γ in the lungs was surprisingly low, and varied during the course of infection, with the highest responses during the acute stage and the lowest responses observed as infection reached the chronic stage. These reduced responses occur despite the presence of similar numbers of bacteria in the lungs during these stages of infection. To further understand the underlying mechanisms of the low frequency of effector T cell activation in the lungs, we quantitated CD4^+^ effector T cell responses to the peptide 25 epitope of *M. tuberculosis* Ag85B, a secreted protein targeted by a large number of *M. tuberculosis*-specific CD4^+^ T cells [45]. Ag85B is targeted by 5 of the 9 novel tuberculosis vaccine candidates currently in clinical trials [46], thus understanding its behavior and responses to it in vivo has considerable importance for TB vaccine development. The reduced expression of *fbpB* we observed is consistent with regulation by the state of bacterial growth, though it may be indirectly triggered by the onset of Th1 immunity, since expression of *fbpB* is maintained in mice lacking IFN-γ [38]. Because Ag85B is a cell wall biosynthesis enzyme, down-regulation of *fbpB* has been interpreted as a consequence of transition by *M. tuberculosis* into a relatively stationary state. Alternatively, *fbpB* suppression during chronic infection may also be an evolved bacterial immune evasion mechanism that enables long-term persistence of *M. tuberculosis* by limiting T cell activation. In support of this, we found that forced expression of *fbpB* by the CPE85B strain during chronic infection resulted in a higher proportion of P25TCRTh1 cells producing IFN-γ than in H37Rv-infected mice. Other studies have suggested but not directly examined the possibility that over-expression of certain *M. tuberculosis* proteins (including Hsp70 and ESAT-6) may cause attenuation of bacterial persistence by increased immune recognition [47], [48]. Our finding that polyclonal CD4^+^ effector T cell responses diminish in chronic infection suggests that this may be a general phenomenon in tuberculosis. Importantly though, the higher frequency of P25TCRTh1 cell activation observed in CPE85B-infected mice diminished at a later time point as it did in H37Rv infection, implying that other mechanisms, especially impairment of MHC II antigen presentation by *M. tuberculosis*, exist to limit effector T cell activation during chronic infection in vivo.

Several in vitro studies have found that *M. tuberculosis* subverts or impairs antigen presentation by the cells it infects, limiting the capability of infected APCs to activate antigen specific T cells [8], [10]. Initial observations include the finding that *M. bovis* BCG survives in primary human macrophages that CD4^+^ T cells fail to recognize [26] and that *M. tuberculosis*-infected THP-1 cells express low amounts of surface MHC II [25]. Several mechanisms for inhibition of MHC II antigen presentation have been characterized using a spectrum of mycobacterial strains and cell components. Among these, impaired phagosome maturation, a well-characterized component of the ability of *M. tuberculosis* to survive in phagocytic cells [19], has been found to limit activation of cathepsin D for efficient processing of mycobacterial antigens [24], while inducing autophagy with rapamycin was recently found to improve the efficacy of BCG and other live mycobacterial vaccines, by enhancing presentation of mycobacterial antigens [49]. Impaired expression of MHC II by macrophages after IFN-γ treatment was also observed after in vitro infection or treatment of macrophages with certain mycobacterial cell components [23], [50], [51], [52], [53]. This effect may involve prolonged signals received through bacterial pattern recognition receptors (PRRs) including TLR2, although we recently reported a TLR2 independent mechanism for impaired MHC II expression in response to IFN-γ [53], [54].

These and other in vitro studies are consistent with our present results and lend support for the hypothesis that APCs do not efficiently stimulate CD4^+^ effector T cells in the lungs during *M. tuberculosis* infection in vivo. Attempts to verify and explore the significance of these in vitro findings with in vivo infection models have been limited thus far, until the present paper. One study of mouse infection with GFP-expressing *M. bovis* BCG found a modest decrease in surface expression of MHC II on some populations of lung APC that harbored intracellular bacteria when compared to those that did not contain bacteria [55]. In contrast, in a low dose aerosol infection of mice with GFP-expressing H37Rv, we did not detect a difference in surface MHC II expression between infected and non-infected APCs at various time points post-infection; we also found that *M. tuberculosis*-infected APCs isolated from the lungs expressed high levels of the costimulatory molecules CD80 and CD86 [54]. Nonetheless, there is evidence that the activation of *M. tuberculosis*-specific T cell responses is impaired during in vivo infection, indicating that *M. tuberculosis* may specifically impair presentation of its antigens without decreasing overall surface expression of MHC II. One recent study found that mice provided with CD4^+^ TCR-transgenic effector T cells specific for the *M. tuberculosis* antigen ESAT-6 prior to infection can restrict bacterial population size to a lower level but cannot prevent establishment of infection [56]. Despite the presence of this effector T cell population in the lungs from the onset of infection, control of bacterial growth was delayed until 7 days post-infection. Likewise, despite mounting apparently normal anti-*M. tuberculosis* CD4^+^ T cell responses, infected mice and humans treated with anti-mycobacterial drugs to eliminate primary infection remain susceptible to reinfection [33], [57]. These studies indicate that susceptibility to persistent tuberculosis is more likely due to failure to activate antigen-specific effector T cells, rather than to insufficient development of antigen specific T cells in response to infection.

We observed increased survival of wild type, but not CD4^+^ T cell-deficient mice infected with the CPE85B strain when compared to those infected with H37Rv, highlighting the importance of enhanced T cell stimulation to the long-term outcome of infection, and indicating that enhanced effector T cell activation, through increased antigen availability, can be accomplished without detrimental effects. Moreover, our finding that sustained expression of Ag85B during the adaptive immune phase of infection was associated with a 2- to 5-fold increase in antigen-specific CD4^+^ T cell activation, yet reduced the bacterial burdens approximately 10-fold implies that a massive increase in effector T cell activation is not necessary to significantly improve immune control of tuberculosis. Future efforts to develop tuberculosis therapies should therefore aim to bypass or overcome factors that limit effector T cell activation including direct T cell suppression, impaired antigen presentation, and bacterial gene regulatory mechanisms. For example, we found that the chronic phase antigen deficit resulting from bacterial suppression of *fbpB* could be overcome by systemic treatment of infected mice with synthetic peptide 25, which strongly but transiently enhanced CD4^+^ T cell responses specific for this epitope and reduced the bacterial burden. This result implies that the endogenous CD4^+^ T cells generated in response to infection with *M. tuberculosis* and recruited to the infected lungs can be stimulated to perform their effector functions if they are provided antigen, resulting in improved bacterial clearance (Figure 7B). The potential for anti-tuberculosis therapies that aim to enhance existing T effector cell responses in infected individuals with synthetically produced peptides encoding known T cell epitopes remains unexplored; however, given the steadily increasing prevalence of drug resistant *M. tuberculosis*, such immunotherapeutic approaches to tuberculosis are an attractive option. Although the consequences of increasing the activation of existing T cell responses have not been widely tested, in the context of certain highly monoclonal T cell responses, administration of epitope peptides has caused rapid mortality of infected or previously immunized mice [58], [59]. However, despite these findings and concerns about possible immunopathology induced by hyperactivation of effector T cells in tuberculosis [60], we observed no morbidity or mortality in infected mice repeatedly treated with peptide 25, a result that encourages the continued exploration of this therapeutic strategy. Future studies should also aim to determine the host and bacterial regulatory mechanisms that account for chronic phase suppression of *fbpB* and whether genes encoding other immunodominant *M. tuberculosis* antigens behave similarly. Identification of the elements of this host-pathogen interaction may lead to the development of therapies that target antigen gene suppression and inhibition of antigen presentation and provide a novel strategy for overcoming bacterial persistence in vivo, leading to better outcomes in *M. tuberculosis*-infected individuals.

## Methods

### Mice

C57BL/6, B6.SJL-Ptprc^a^ Pepc^b^/BoyJ (CD45.1^+^), and MHCII KO mice for aerosol *M. tuberculosis* infection experiments were either bred in the New York University School of Medicine Skirball animal facility or purchased from Taconic Farms, Inc. P25TCR-Tg mice, whose CD4^+^ T cells express a transgenic T-cell antigen receptor that recognizes the complex of peptide 25 (aa 240–254) of *M. tuberculosis* Ag85B and the mouse MHC II allele I-A^b^ were prepared on a C57BL/6 background, as previously described [2], [61]. All animal experiments were done in accordance with procedures approved by the NYU School of Medicine Institutional Animal Care and Use Committee and in strict accordance with the recommendations in the Guide for the Care and Use of Laboratory Animals of the National Institutes of Health under the Assurance of Compliance Number A3435-01.

### *M. tuberculosis* in vitro growth and aerosol mouse infection

Wild type *M. tuberculosis* H37Rv was originally obtained from ATCC. Frozen stocks for aerosol infection and in vitro use were prepared and stored at −80°C. GFP-expressing H37Rv and Ag85B null (ΔAg85B) strains of *M. tuberculosis* were generated as previously described [2], [62]. *M. tuberculosis* cultures were grown in 10 mL Middlebrook 7H9 liquid medium supplemented with 10% v/v albumin dextrose catalase enrichment and incubated under shaking conditions at 37°C. Mice at 8–12 weeks of age were infected with ∼100 CFU of *M. tuberculosis* via the aerosol route using an Inhalation Exposure Unit (Glas-Col) as previously described [62]. To verify inoculum size, 3–5 infected mice were euthanized 24 hours after infection and lungs were homogenized and plated on Middlebrook 7H11 medium supplemented with 10% v/v albumin dextrose catalase enrichment. To determine bacterial population size at time points post-infection, lungs were homogenized, diluted in PBS+Tween-80 (0.5%), and added to 7H11 plates. Plates were incubated at 37°C for 3 weeks and single colonies were counted. To determine *M. tuberculosis* survival in stationary culture, 7H9 medium was inoculated with H37Rv or CPE85B, grown in shaking conditions to saturation (O.D._600_>1.0), and initial CFUs were measured. Cultures were then placed in stationary incubator at 37°C for 17 days, and final CFUs were measured.

### Fluorescent microscopy of frozen tissue sections

C57BL/6 mice were infected with *M. tuberculosis* H37Rv and on day 25 post-infection received 1×10^6^ CFP^+^ P25TCRTh1 cells via adoptive transfer. On 28 post-infection, lungs were perfused and frozen in OCT before 5 µm sectioning and fixation in cold acetone. Sections were stained with DAPI to label nuclei and analyzed on a Leica DMRB fluorescent microscope (objective: Leica PL Fluotar 20×/0.50) equipped with a Spot RT digital camera. Separate images for DAPI and CFP fluorescence were acquired and merged using Spot software.

### P25TCRTh1 CD4^+^ effector T cells

P25 TCR-Tg CD4^+^ Th1 effector cells were generated in vitro as follows: naïve CD4^+^ T cells were magnetically isolated from lymph node cell suspensions of P25 TCR-Tg mice (or for fluorescent microscopy, a P25TCR-Tg mouse expressing CFP under control of the ubiquitin promoter) using CD4 (L3T4) microbeads and an AutoMACS (Miltenyi Biotech). P25TCR-Tg CD4^+^ T cells were co-cultured with irradiated C57BL/6 splenocytes in the presence of mouse IL-12p70 (10 ng/ml), mouse IL-2 (5 ng/ml), anti-IL-4 neutralizing antibody (50 ng/ml), and synthetic peptide 25 (0.5 µM). Cells were cultured at 37°C with 5% CO_2_. On days 3 and 5 of culture, cells were split 1∶3 with fresh media containing IL-12p70, IL-2, and anti-IL-4, but no peptide 25. Cells were washed with PBS and counted on day 7 of culture before use for in vitro or in vivo assays. For in vitro restimulation, P25TCRTh1 cells were co-cultured with irradiated C57BL/6 splenocytes for 24 hours in RPMI-10 in the presence or absence of peptide 25 (0.5 µM) or bone marrow derived dendritic cells infected with *M. tuberculosis* (MOI: 0.1). Cells were collected and analyzed by flow cytometry for intracellular IFN-γ, or culture supernatants were analyzed for IFN-γ by ELISA. For in vivo experiments, 1×10^6^ P25TCRTh1 cells were injected via tail vein or retro-orbital sinus into recipient mice at various time points post-infection. Cells were routinely isolated from lungs of recipient mice 72 hours after adoptive transfer and analyzed by flow cytometry.

### Naïve P25TCR-Tg T cell proliferation

3×10^6^ CFSE-labeled CD4^+^ T cells, harvested from the lymph nodes of P25TCR-Tg mice were adoptively transferred into infected recipients at various time points post-infection. 7 days after adoptive transfer, mediastinal lymph nodes were harvested from recipient mice and cells were analyzed for CFSE dilution by flow cytometry.

### Generation of CPE85B strain of *M. tuberculosis*

The Ag85B null strain of *M. tuberculosis* (ΔAg85B), previously created by our lab from wild-type H37Rv [2], was used as a background strain for generating CPE85B. Both the *hspX* promoter sequence, consisting of 254 bp directly 5′ of the *hspX* start codon, as well as the *fbpB* open reading frame were amplified by PCR from H37Rv genomic DNA. Each of these fragments was ligated into the pMV306 integrating vector to create a recombinant construct, whose sequence was verified by Sanger sequencing performed by the NYU DNA sequencing facility. ΔAg85B was grown in 7H9 liquid media and transformed with this construct via electroporation. The reaction was plated on 7H11 plates containing 25 µg/ml kanamycin to select for bacteria incorporating the construct into the *M. tuberculosis* chromosome. Presence of the construct in kanamycin resistant colonies was verified by PCR. Expression and secretion of Ag85B by CPE85B was confirmed by SDS-PAGE and anti-Ag85B western blot of supernatants from 7H9 liquid medium after stationary culture. For stationary culture-induced expression of Ag85B by the CPE85B strain, 10 mL cultures were grown to late phase (OD_600_∼1.0) in normal shaking conditions, then flasks were sealed and transferred to a stationary incubator for >1 week before supernatants were collected.

### RT-qPCR of bacterial mRNA from infected mouse lungs

To quantitate expression of *M. tuberculosis* genes during mouse infection, lungs of infected mice were rapidly placed into a solution of RNAlater (Ambion) and stored overnight at room temperature in accordance with manufacturer recommendations to allow permeation of the tissue. Thereafter, samples for RNA isolation were stored at −80°C. When comparing expression of genes at various time points, tissues were transferred to TRIzol (Invitrogen) and quickly homogenized using a Tissue Tearor homogenizer to disrupt mouse cells. Lung homogenates were centrifuged to pellet intact bacterial cells, and supernatants discarded. *M. tuberculosis* pellets were disrupted with zirconia/silica beads, RNA was extracted, and RT-qPCR was carried out as previously described [37] with *fbpB* copy number normalized to the constitutively expressed 16S rRNA and multiplied by a factor of 10^5^. The following RT-qPCR primers were used in this study. 16S rRNA: RT 5-ATTACGTGCTGGCAACATGA-3, qPCR For 5-GCCGTAAACGGTGGGTACTA-3, qPCR Rev 5-TGCATGTCAAACCCAGGTAA-3; *hspx/acr/Rv2031c*: RT 5-GAATGCCCTTGTCGTAGGTG-3, qPCR For 5-AGATGAAAGAGGGGCGCTAC3, qPCR Rev 5-TAATGTCGTCCTCGTCAGCA3; *fbpB/Rv1886c*: RT 5-TCCTGGAACTTCAGGTTGCT-3, qPCR For 5-ACCCCCAGCAGTTCATCTAC-3, qPCR Rev 5-TTCCCGCAATAAACCCATAG-3.

### Tissue processing and flow cytometry

To isolate cells from infected tissues for flow cytometry, mice were euthanized with CO_2_ followed by cervical dislocation. Tissues were removed and mechanically disrupted by mincing in RPMI as previously described [62] or using a gentleMACS dissociator (Miltenyi Biotec) in the manufacturer-recommended HEPES buffer. Lung suspensions were incubated in Collagenase D and DNase at 37°C with 5% CO_2_ for 30 minutes and cells were isolated by forcing suspensions through a 70 µM cell strainer. RBCs were removed by ACK lysis and live cells counted by trypan blue exclusion. Cell suspensions were stained using the following fluorescently-labeled antibodies (Biolegend, BD Pharmingen, or eBioscience): anti-CD3 PE, anti-CD4 (L3T4) FITC, anti-CD45.2 PerCP, anti-CD45.1 Pacific Blue, anti-IFN-γ (XMG1.2) APC, and rat IgG1 APC isotype control. Flow cytometry was performed using a FACSCalibur or LSR II (BD Biosciences) at the NYU Cancer Institute Flow Cytometry and Cell Sorting facility. Analysis of flow cytometry data was performed using FlowJo software.

### Detection of IFN-γ-producing cells by direct intracellular cytokine staining

To detect intracellular IFN-γ produced by cells in vivo, a protocol was developed based on a previous study [28]. In contrast to this study, however, optimal detection of IFN-γ producing cells from the lungs of mice infected with *M. tuberculosis* did not require treatment of mice with i.v. brefeldin A or inclusion of brefeldin A in tissue processing buffers. Instead, after euthanasia, tissues were rapidly placed on ice and all cell isolation steps except collagenase/DNase digestion (37°C for 30 minutes) and ACK lysis (room temperature for 5 minutes) were carried out quickly and on ice. Cells were stained for surface markers at 4°C for 30 minutes followed by permeabilization and fixation with Cytofix/Cytoperm (BD Biosciences) at 4°C for 20 minutes. Finally, fixed cells were stained with anti-IFN-γ or a rat IgG1 isotype control at 4°C for 30 minutes. Flow cytometry dot plot gates for IFN-γ^+^ cells were set based on comparison with isotype control and unpermeabilized cells stained for IFN-γ.

### Total CD4^+^ T cell depletion

Mice were treated with an intra-peritoneal dose of 500 µg of either monoclonal antibody GK1.5, which depletes CD4^+^ T cells, or a rat IgG2b isotype control (LTF-2) every 6 days from day 28 to Day 50 post-infection. Efficiency of CD4^+^ T cell depletion 6 days after GK1.5 treatment was determined to be >95% by flow cytometry of cell suspensions from lungs, spleen and blood. In mice treated with LTF-2 isotype control, no differences were observed in CD4^+^ T cell number or bacterial burden when compared to untreated mice.

### Selective ablation of endogenous CD4^+^ T cells

To determine the influence of endogenous CD4^+^ T cells on the response of adoptively transferred P25TCRTh1 cells in vivo, a system was developed to deplete endogenous CD4^+^ T cells selectively from infected mice. Mice expressing Cre recombinase under control of the CD4 promoter were crossed with those carrying an inducible Diphtheria Toxin Receptor (iDTR) allele, whose baseline expression is prevented by a stop codon flanked by *loxp* sites [34]. Progeny of this cross (CD4-DTR) carry CD4^+^ T cells that are sensitive to Diphtheria Toxin mediated ablation. CD4-DTR mice were infected with H37Rv and received daily intraperitoneal doses of DT (100 ng) to ablate endogenous CD4^+^ T cells from day 21 to day 28 post-infection. The efficiency of CD4^+^ T cell ablation in the lungs was determined by flow cytometry to be 48.9%. P25TCRTh1 cells were adoptively transferred on day 25 post-infection and the frequency of IFN-γ production was assessed on day 28 post-infection.

### Assessment of intravascular and extravascular location of adoptively-transferred P25TCRTh1 cells

On day 25 post-infection, wild-type mice infected with *M. tuberculosis* H37Rv received P25TCRTh1 cells via adoptive transfer. On day 28 post-infection, mice were treated intravenously with 800 ng (at 4.0 ng/µL) PerCP-labeled anti-CD4 (RM4-5). Fifteen minutes later, mice were euthanized and total lung cells were stained with FITC-labeled anti-CD4 (GK1.5). Lung cells stained by anti-CD4-PerCP were considered to be CD4^+^ T cells residing in the intravascular compartment at the time of antibody injection. Cells staining positive for anti-CD4-FITC and negative for PerCP were considered to be CD4^+^ T cells residing in an extravascular or parenchymal lung compartment protected from labeling with intravenous antibody. IFN-γ production in vivo was assessed by intracellular staining of all cells with APC-labeled anti-IFN-γ as previously described.

### Systemic treatment of mice with synthetic peptides

Mice were intravenously treated with 100 µg of Ag85B peptide 25 (FQDAYNAAGGHNAVF) or OVA peptide control (ISQAVHAAHAEINEAGR) in 100 µl sterile PBS via tail vein or retro-orbital sinus. Peptides were synthesized by EZBiolab or Peptides International to a purity of >95%.

### Statistical analyses

Data shown are representative of 2 or more experimental replicates. In all figures, error bars indicate mean ± SEM. To determine statistical significance when comparing experimental values from two groups of mice, one- or two-tailed student's *t*-tests were routinely used, each where appropriate. To compare the growth rate of H37Rv and CPE85B in vivo, a non-linear regression analysis (curve fit) with F-test was used to determine whether a single curve could account for both data sets. In mouse survival experiments, Logrank test was used to evaluate statistical significance when comparing survival of one mouse strain after infection with either of the two bacterial strains. * = p<0.05; ** = p<0.005; n.s = not significant.

## Supporting Information

Figure S1 Brefeldin A treatment does not improve detection of IFN-γ produced by CD4^+^ cells in vivo. Frequency of lung CD4^+^ T cells on day 28 post-infection that stain with anti-IFN-γ antibody or isotype control. Mice infected with *M. tuberculosis* were treated with 250 µg intravenous brefeldin A or left untreated. 6 hours after treatment, lungs were processed on ice in buffer alone or in buffer containing brefeldin A (20 µg/mL). Flow cytometry plots show lung CD4^+^ cells from a representative mouse in two experiments with n = 3 mice. Values indicate the proportion of IFN-γ^+^ cells among CD4^+^ population for each mouse.
(TIF)

Figure S2 Adoptively transferred P25TCRTh1 cells efficiently enter lung parenchyma and produce IFN-γ. A. The percentage of P25TCRTh1 cells adoptively transferred into infected mice that stain PerCP^−^ or PerCP^+^ after intravenous treatment with PerCP-labeled anti-CD4. Histogram gates indicate the fraction of CD4^+^, CD45.2^+^ lung cells that are either PerCP^−^ (parenchymal) or PerCP^+^ (intravascular). B. The fraction of P25TCRTh1 cells from parenchymal (left, PerCP^−^) or intravascular (right, PerCP^+^) compartments that are activated in vivo to produce IFN-γ.
(TIF)

Figure S3 Ablation of endogenous CD4^+^ T cells does not affect IFN-γ production by adoptively transferred P25TCRTh1 cells. A. The frequency of (CD45.2^+^) endogenous CD4^+^ T cells among total lung cells from CD4-DTR mice 28 days after infection. Mice left untreated or were treated daily for 7 days prior to analysis with diphtheria toxin to ablate endogenous CD4^+^ T cells. B. The effect of endogenous CD4^+^ T cell ablation on the fraction of (CD45.1^+^) P25TCRTh1 cells adoptively transferred on day 25 post-infection that are activated in the lungs to produce IFN-γ.
(TIF)
