# Supplementary figures and images for: Suboptimal Activation of Antigen-Specific CD4+ Effector Cells Enables Persistence of M. tuberculosis In Vivo
Source: PLoS Pathog. 2011 May 26;7(5):e1002063. doi: 10.1371/journal.ppat.1002063 (PMC3102708; doi:10.1371/journal.ppat.1002063)

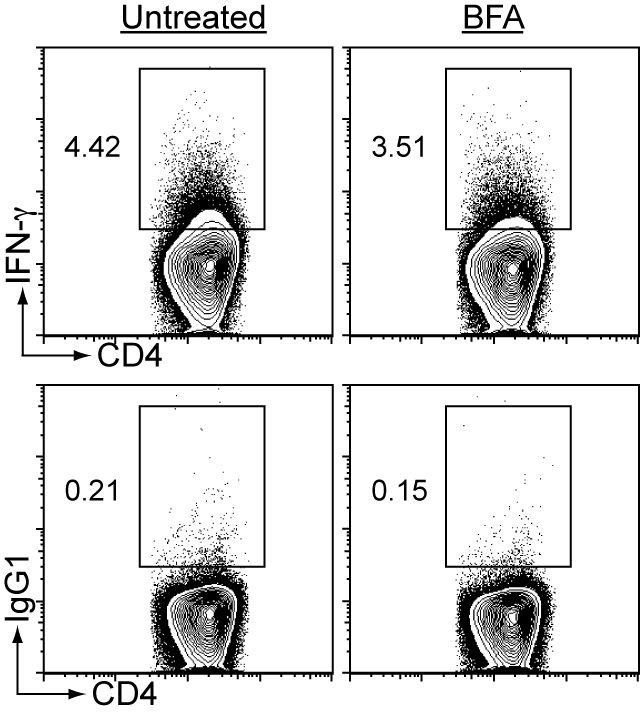

Supplement: Figure S1 — Brefeldin A treatment does not improve detection of IFN-γ produced by CD4+ cells in vivo. Frequency of lung CD4+ T cells on day 28 post-infection that stain with anti-IFN-γ antibody or isotype control. Mice infected with M. tuberculosis were treated with 250 µg intravenous brefeldin A or left untreated. 6 hours after treatment, lungs were processed on ice in buffer alone or in buffer containing brefeldin A (20 µg/mL). Flow cytometry plots show lung CD4+ cells from a representative mouse in two experiments with n = 3 mice. Values indicate the proportion of IFN-γ+ cells among CD4+ population for each mouse. (TIF) [file ppat.1002063.s001.tif]

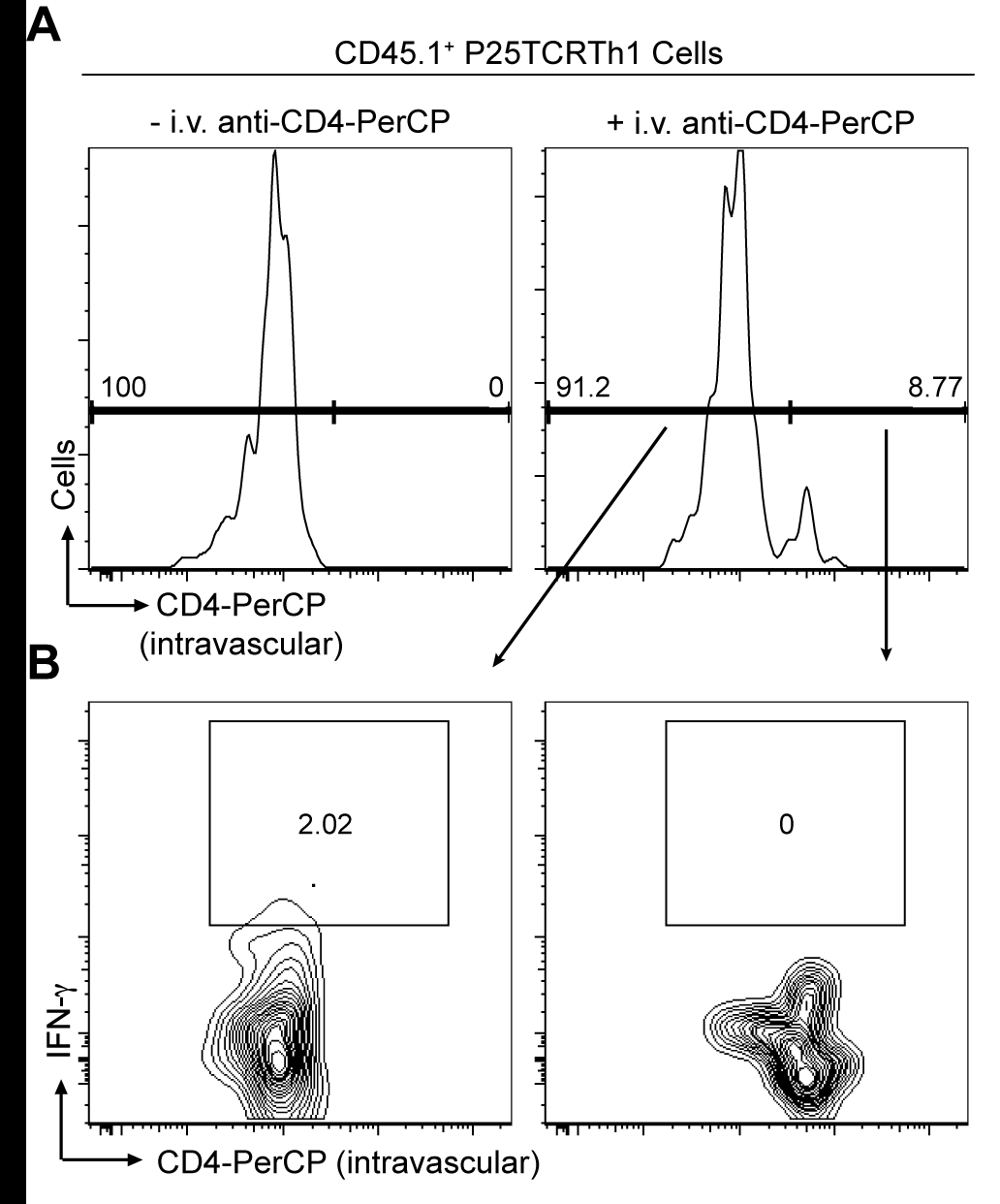

Supplement: Figure S2 — Adoptively transferred P25TCRTh1 cells efficiently enter lung parenchyma and produce IFN-γ. A. The percentage of P25TCRTh1 cells adoptively transferred into infected mice that stain PerCP− or PerCP+ after intravenous treatment with PerCP-labeled anti-CD4. Histogram gates indicate the fraction of CD4+, CD45.2+ lung cells that are either PerCP− (parenchymal) or PerCP+ (intravascular). B. The fraction of P25TCRTh1 cells from parenchymal (left, PerCP−) or intravascular (right, PerCP+) compartments that are activated in vivo to produce IFN-γ. (TIF) [file ppat.1002063.s002.tif]

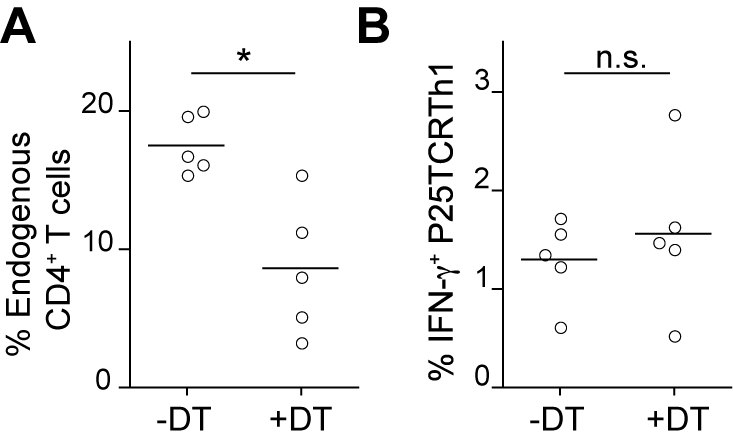

Supplement: Figure S3 — Ablation of endogenous CD4+ T cells does not affect IFN-γ production by adoptively transferred P25TCRTh1 cells. A. The frequency of (CD45.2+) endogenous CD4+ T cells among total lung cells from CD4-DTR mice 28 days after infection. Mice left untreated or were treated daily for 7 days prior to analysis with diphtheria toxin to ablate endogenous CD4+ T cells. B. The effect of endogenous CD4+ T cell ablation on the fraction of (CD45.1+) P25TCRTh1 cells adoptively transferred on day 25 post-infection that are activated in the lungs to produce IFN-γ. (TIF) [file ppat.1002063.s003.tif]
